# Obstructive Sleep Apnea as a Predictor of Arrhythmias in 24-h ECG Holter Monitoring

**DOI:** 10.3390/brainsci11040486

**Published:** 2021-04-12

**Authors:** Dominika Urbanik, Paweł Gać, Helena Martynowicz, Maciej Podgórski, Małgorzata Poręba, Grzegorz Mazur, Rafał Poręba

**Affiliations:** 1 Department of Internal Medicine, Occupational Diseases, Hypertension and Clinical Oncology, Wroclaw Medical University, Borowska 213, PL 50-556 Wroclaw, Poland; dominika.urbanik@umed.wroc.pl (D.U.); helena.martynowicz@umed.wroc.pl (H.M.); maciej.podgorski@umed.wroc.pl (M.P.); grzegorz.mazur@umed.wroc.pl (G.M.); rafal.poreba@umed.wroc.pl (R.P.); 2 Department of Hygiene, Wroclaw Medical University, Mikulicza-Radeckiego 7, PL 50-368 Wroclaw, Poland; 3 Department of Pathophysiology, Wroclaw Medical University, Marcinkowskiego 1, PL 50-368 Wroclaw, Poland; malgorzata.poreba@umed.wroc.pl

**Keywords:** obstructive sleep apnea, ECG Holter monitoring, apnea-hypopnea index, supraventricular arrhythmias, supraventricular tachycardia

## Abstract

A relationship between obstructive sleep apnea (OSA) and abnormalities in 24-h electrocardiogram (ECG) Holter monitoring has not been sufficiently documented. The aim of this study was to analyze the relation between the occurrence and the severity of OSA and the parameters of ECG Holter monitoring in a group of patients with diagnosed OSA. Ninety-four patients with clinical suspicion of OSA were qualified for the study (mean age 53.7). All participants underwent a medical history, laboratory tests, 24-h ECG Holter monitoring, and single-night video-polysomnography (vPSG) using the American Academy of Sleep Medicine recommendations. A group of patients with diagnosed OSA was characterized by more frequent occurrence of supraventricular tachycardia (SVT) and ventricular arrhythmias (VPC). A statistically significant positive correlation was shown between the apnea-hypopnea index (AHI) and average heart rate, supraventricular arrhythmias (SVPC) pairs, SVT, and pauses >2.5 s. In regression analysis, higher AHI constituted an independent predicator for the increased number of pauses >2.5 s, SVT, and SVPC pairs in 24-h ECG Holter monitoring. In summary, patients with OSA are characterized by the increased number of abnormalities in 24-h ECG Holter monitoring.

## 1. Introduction

Obstructive sleep apnea (OSA) is a multifactorial disease that is an increasing social problem of the 21st century. OSA occurs in about 5% of women and 14% of men. It may affect any age group, but most frequently middle age men suffer from this disease [1]. In some populations, prevalence of OSA is significantly higher, reaching up to 70% in the group of patients treated bariatrically and after a stroke [2,3]. OSA is characterized by recurrent episodes of collapse of the upper respiratory tract resulting in limited or completely blocked air flow (clinically, hypopnea, and apnea are observed). Respiratory events also include respiratory effort related arousal (RERA) [4]. The most frequent causes of sleep apnea are obesity and an enlarged neck circumference; other factors also include laryngological diseases such as looseness of the soft palate, tonsil hyperplasia, macroglossia, and excess throat mucus [5], and craniofacial disorders such as retrognation and micrognathism [6,7].

Sleep apnea constitutes an independent risk factor for cardiovascular diseases, in particular hypertension, ischemic heart disease, strokes, heart failure, atrial fibrillation, and sudden cardiac death [8]. Pathogenesis of cardiovascular disease development in patients with sleep apnea is not fully known; most likely it has a multifactorial nature, including excessive activation of inflammatory factors, endothelial dysfunction, increased blood clotting, metabolic disorders, and activation of the sympathetic nervous system [9,10]. Recurrent episodes of hypoxemia, respiratory acidosis, and nocturnal arousals result in the disturbance of the autonomic nervous system balance. Domination of the sympathetic nervous system favors development of arrhythmias which constitute an important, often underestimated problem in epidemiology of cardiovascular diseases [11].

A relationship between incidence, as well as severity of OSA, and 24-h electrocardiogram (ECG) Holter abnormalities has not been sufficiently documented. The significance of co-occurrence of other cardiovascular diseases and cardiovascular risk factors on the implicit relationship between OSA and arrhythmias seems to be interesting.

The purpose of this study was to analyze the relationships between the occurrence and the severity of OSA and the parameters of ECG Holter monitoring in a group of patients with diagnosed OSA.

## 2. Materials and Methods

Patients were enrolled between October 2017 and September 2018 in the Sleep Laboratory of the Department of Internal Medicine, Occupational Diseases, Hypertension and Clinical Oncology at the Wroclaw Medical University. The exclusion criteria were as follows: inability to undergo polysomnography, presence of severe mental disorders, active malignancy, coexistence of respiratory insufficiency, or active inflammation. The inclusion criteria included the following: willingness to participate in the study, aged above 18 years, and clinically suspected OSA. Ninety-four patients with clinical suspicion of obstructive sleep apnea hospitalized in the department of internal medicine were finally qualified for the study. Five people were excluded from the study, in the case when polysomnographic record and ECG Holter monitoring was not recorded for at least 6 h. The remaining group of 89 patients, including 51 men and 38 women, was considered to be the representative sample. The average age of the participants was 53.7 (range 24–72). In most participants, obesity (62.9%) and hyperlipidemia (79.8%) were diagnosed; 22.5% of the participants had diabetes, 85.4% hypertension, 11.2% coronary disease, and 3.4% reported stroke in their past. Active smokers constituted 40.4% of the participants (Table 1).

For statistical purposes, patients were divided into a group of patients with OSA (group A) and a group of healthy patients (group B). Subsequently, the following subgroups were chosen considering the apnea-hypopnea index (AHI) threshold value: subgroup A1 with AHI ≥ 15 (*n* = 55), and A2 with AHI < 15 (*n* = 34), and then subgroup A3 with AHI ≥ 30 (*n* = 30), and subgroup A4 with AHI < 30 (*n* = 59).

The study was approved by the Bioethics Committee of the Wroclaw Medical University. Informed consent was obtained from all individual participants included in the study. All participants underwent a medical history, laboratory tests, 24-h ECG Holter monitoring, and single-night video-polysomnography (vPSG). The study concerned demographic and anthropometric data (age, gender, weight, and height), cardiovascular risk factors in history (smoking, hypertension, hyperlipidemia, and diabetes) and cardiovascular diseases (ischemic heart disease and stroke).

Fasting total cholesterol concentration as well as LDL and HDL cholesterol, triglycerides, and glucose levels in blood were tested. Commercially available tests were used.

In all patients, 24-h ECG Holter monitoring was carried out. The ECG Holter recordings were made using a 12-channel Lifecard CF and the analysis was performed using the Sentinel Spacelabs Healthcare Pathfinder SL (Delmar Reynolds, Hertford, Great Britain). During Holter monitoring, patients had normal daily activities and their activities were recorded in diaries. Patients were instructed to follow the established hours of daily activities (6:00–22:00) and night sleep (22:00–6:00) during ECG monitoring. All Holter monitoring ECG records were analyzed by one person. During the analysis of ECG records, the person assessing them did not have any clinical data of the patients, in particular, the person did not have any information concerning the incidence and severity of OSA.

Qualitative and quantitative Holter parameters were assessed. Among qualitative parameters, the origin of rhythm was considered (sinus rhythm, escape rhythm, and atrial fibrillation), presence of ventricular tachycardia (VT), tachycardia, bradycardia, pause >2.5 s, I-, II-, and III-degree atrioventricular block, and ST-T changes. Quantitative parameters included mean, minimum and, maximum heart rate (HR); the number of arrhythmias in the form of single premature supraventricular arrhythmias (SVPC) and ventricular arrhythmias (VPC); SVPC pairs; ventricular trigeminy and bigeminy; and episodes of supraventricular tachycardia (SVT), tachycardia, bradycardia, and pauses >2.5 s.

Each patient from the study group was subjected to complete single full-night polysomnography using the NoxA1 device (Nox Medical, Reykjavik, Iceland) in the sleep laboratory. Records from numerous sensors were considered in the analysis. Bioelectric activity of the brain (electroencephalogram, EEG) was registered as well as eye movements (electrooculogram, EOG), muscle tension of chin (electromyogram, EMG). Nasal pressure transducer was used to assess hypopnea and oronasal thermal airflow sensor was used to assess apnea. The movements of the chest and abdomen were assessed using inductive plethysmography. The pulse and level of saturation data were recorded using a NONIN WristOx2 3150 pulse oximeter (Nonin Medical, Inc., Plymouth, MN, USA). EEGs were recorded using the American Academy of Sleep Medicine (AASM) recommended EEG montages. A certificated and experienced physician (H.M.) scored and analyzed the PSG recordings adhering to the AASM guidelines [12].

Apnea was coded at airflow reduced by ≥90% from the thermistor sensor lasting ≥10 s. Hypopnea was defined as a reduction in air flow from the pressure sensor by more than 50% as compared with the basic record which took ≥10 s with accompanying desaturation ≥3% or arousal. The AHI (apnea-hypopnea index) was considered in the analysis of the results. The AHI values for adults are categorized as follows: normal (AHI < 5 events/h), mild sleep apnea (AHI 5–15 events/h), moderate sleep apnea (AHI 16–30 events/h), and severe sleep apnea (AHI ≥ 30 events/h). Polysomnography and ECG Holter monitoring were conducted simultaneously.

### Statistical Methods

The statistical analysis was based on the statistical program STATISTICA 13 (StatSoft Poland). Means and standard deviations were calculated for quantitative variables. The distribution of variables was checked with the Shapiro–Wilk W test. Due to the lack of a normal distribution of variables, testing of hypotheses concerning quantitative variables was performed with the Mann–Whitney U test. Qualitative variables were presented as percentages. The chi-square test with the highest reliability was used to test hypotheses concerning qualitative variables. The relationships between the studied variables were assessed using correlation and regression analysis. Spearman’s R correlation analysis was used. Regression analysis models were estimated using the least squares method. The level of statistical significance was set at *p* < 0.05.

## 3. Results

A group of patients with diagnosed sleep apnea (group A) is characterized by more frequent occurrence of SVT and VPC arrhythmias (Table 2).

The analysis of qualitative parameters in groups A and B did not indicate any statistically significant differences (Table 3).

Comparison of types of arrhythmias separately for subgroup A1 and A2 as well as subgroup A3 and A4 was carried out. It was indicated that patients with AHI ≥ 15 (subgroup A1) have a higher average and minimum heart rate and more frequent premature ventricular complexes (VPC) than patients without apnea and with mild apnea. In the subgroup of patients with severe apnea (subgroup A3) a higher incidence of VPC was found in comparison to other patients (Table 4).

The analysis of qualitative parameters indicated statistically significant differences for pauses and 1st degree atrioventricular block in subgroups A3 and A4. The abovementioned conduction abnormalities occur more often in patients with severe apnea (Table 5).

A statistically significant positive correlation was shown between AHI and HR mean (*r* = 0.22, *p* = 0.041), SVPC pairs (*r* = 0.40, *p* = 0.001), SVT (*r* = 0.23, *p* = 0.032), and pauses >2.5 s (*r* = 0.24, *p* = 0.025).

In the next stage, the relationships between AHI and HR mean, AHI and SVPC pairs, AHI and SVT, as well as AHI and pauses >2.5 s were analyzed by regression analysis for the impact of potential modifying variables. The variables potentially related to the parameters of the 24-h Holter monitoring ECG record were gender, age, BMI, total cholesterol, HDL cholesterol, triglycerides, glucose, diabetes mellitus, arterial hypertension, coronary artery diseases, stroke, smoking, and AHI. In the study group, independent predictors of higher HR mean were smoking, more SVPC pairs were higher AHI and higher BMI, more SVTs were higher AHI and male gender, and more pauses >2.5 s were higher AHI and higher glucose concentration (Table 6).

## 4. Discussion

Reviews of previous studies have shown that sleep apnea constitutes a significant risk factor for development of arrhythmias. Data from the Busselton Health Study showed that moderate and severe apnea were related to increased overall mortality regardless of the cause [13]. The same results were obtained by T. Young in his Wisconsin Sleep Cohort study which additionally stated that treatment with continuous positive airway pressure (CPAP) reduced the risk of mortality [14]. Another cohort study conducted on 10,701 patients who were observed for five years indicated a correlation between OSA and occurrence of sudden cardiac death (SCD). Gami et al. found that the most important predictive factors for SCD included age >60, AHI > 20, average saturation at night <93%, and the lowest saturation at night <78% [15]. A potential mechanism connecting apnea with the occurrence of sudden cardiac death is an increased QT interval dispersion that induces severe ventricular arrhythmias [16]. In a group of 472 patients with heart failure and an implanted cardiac resynchronization therapy defibrillator (CRT-D), it was shown that apnea was an independent risk factor for ventricular arrhythmia requiring implantable cardioverter-defibrillator (ICD) impulse therapy [17].

Mechanisms of arrhythmias in the case of apnea are complex, which results in various types of arrhythmias, i.e., supraventricular, ventricular arrhythmia, and conduction defects. Selim et al. indicated a two times higher risk of arrhythmias during sleep in patients with moderate and severe apnea [18]. In the study conducted by Mehr et al., it was observed that in patients with apnea atrial fibrillation and complex ventricular arrhythmias in the form of bigeminy, trigeminy, and non-sustained ventricular tachycardia occurred more frequently [11]. In a group of 3542 patients, recurrent episodes of desaturation characteristic for OSA turned out to be an independent risk factor for atrial fibrillation in a five-year observation of patients <65 years of age [19]. In patients who underwent pulmonary vein ablation, individuals with moderate and severe apnea had a two times higher risk of the recurrence of atrial fibrillation as compared with a control group without apnea [20]. The relationship between apnea and conduction defects was proven by Becker et al., who indicated correlations between AHI and the presence of pauses, the atrioventricular block, and bradycardia [21]. In a study conducted by Roche, in a group of patients with apnea and coexisting pauses observed for one year of treatment using a device with CPAP, no arrhythmias requiring implantation of a pacemaker were found [22].

In the present study, in which we used highly specialized devices and objective measurement methods, a significant impact of sleep apnea on the induction of mild arrhythmias in the studied patients was observed in the form of the increased number of episodes of supraventricular tachycardia and additional single ventricular complexes in 24-h ECG Holter recording. A higher AHI index was the independent risk factor that predicted the increased number of supraventricular arrhythmias and it significantly impacted the occurrence of conduction defects. A positive correlation between the AHI value and the average heart rate was observed. Considering the obvious influence of OSA on the cardiovascular system, the presence of apnea should be included in the set of diagnostic tests used for SCD risk stratification.

The unquestionable advantages of our study include the blinded analysis of arrhythmias, the independence of the polysomnography results, the assessment of the ECG Holter monitoring with all 12 leads, rigorous and unified collection of survey data on cardiovascular risk; and the performance of additional screening laboratory tests to detect coexisting diseases increasing cardiovascular risk of hyperlipidemia and diabetes.

### 4.1. Limitations

The limitation of this study is the relatively small number of patients. A larger group of patients would probably show the dependency between sleep apnea and the presence of other arrhythmias, such as atrial fibrillation and ventricular tachycardia, which occur less frequently in the general population.

### 4.2. Summary

The conducted study indicates that patients with sleep apnea are burdened with a greater risk of arrhythmias, and therefore most likely higher mortality due to cardiovascular causes. Routine Holter monitoring in patients with OSA would allow for implementation of early prevention and treatment of coexisting cardiac disorders, and therefore a reduction in cardiovascular risk. Taking the prevalence of sleep apnea into account in the general population and the frequent coincidence of OSA with arrhythmias, routine polysomnographic diagnostics in patients with arrhythmias should be considered.

## 5. Conclusions

Patients with OSA are characterized by an increased number of abnormalities in 24-h ECG Holter monitoring. In the study group, a higher AHI constitutes an independent predicator for an increased number of pauses >2.5 s, SVT, and SVPC pairs in 24-h ECG Holter monitoring. The positive linear relation between AHI value and mean HR in ECG Holter recording may be the result of the impact of smoking on heart rate.

## Figures and Tables

**Table 1:** Clinical characteristics of the study group.

	Whole Study Group	OSA (Group A)	Without OSA (Group B)	*p* A-B
Number	89/100.0	77/86.5	12/13.5	-
Men	51/57.3	44/57.1	7/58.3	ns
Women	38/42.7	33/42.9	5/41.7	ns
Age (years)	53.74 ± 12.52	54.58 ± 11.91	48.33 ± 15.39	ns
Height (m)	1.71 ± 0.10	1.71 ± 0.09	1.70 ± 0.11	ns
Body mass (kg)	94.08 ± 20.29	96.54 ± 19.91	78.25 ± 15.39	0.003
BMI (kg/m^2^)	32.00 ± 6.47	32.81 ± 6.42	26.78 ± 3.87	0.001
Obesity	56/62.9	53/68.8	3/25.0	0.003
Total cholesterol (mg/dL)	196.47 ± 44.68	196.54 ± 45.10	196.00 ± 43.80	ns
LDL cholesterol (mg/dL)	114.24 ± 36.19	113.86 ± 36.76	116.91 ± 33.33	ns
HDL cholesterol (mg/dL)	047.61 ± 10.36	47.28 ± 10.54	49.67 ± 9.24	ns
Triglycerides (mg/dL)	185.58 ± 107.20	185.45 ± 101.16	186.42 ± 145.70	ns
Hyperlipidemia	71/79.8	65/84.4	6/50.0	0.006
Glucose (mg/dL)	119.15 ± 42.57	122.13 ± 44.62	100.00 ± 16.67	0.043
Diabetes mellitus	20/22.5	20/25.9	0/0.0	0.045
Arterial hypertension	76/85.4	66/85.7	10/83.3	ns
Coronary artery diseases	10/11.2	9/11.7	1/8.3	ns
Stroke	3/3.4	2/2.6	1/8.3	ns
Smoking	36/40.4	32/41.6	4/33.3	ns
AHI	26.38 ± 23.51	30.07 ± 23.18	2.70 ± 1.43	0.001

OSA, obstructive sleep apnea; AHI, apnea-hypopnea index; LDL, low-density lipoproteins; HDL, high-density lipoproteins; BMI, body mass index.

**Table 2:** Qualitative parameters of 24-h electrocardiogram (ECG) Holter monitoring in the studied group of patients.

	Whole Study Group	OSA (Group A)	Without OSA (Group B)	*p* A-B
HR mean (bpm)	68.91 ± 7.81	69.35 ± 7.63	66.08 ± 8.69	ns
HR min (bpm)	54.39 ± 6.92	54.74 ± 7.02	52.17 ± 6.07	ns
HR max (bpm)	102.65 ± 16.95	102.47 ± 15.45	103.83 ± 25.43	ns
SVPC	146.92 ± 569.19	167.94 ± 609.74	12.08 ± 14.81	ns
SVPC pairs	14.20 ± 106.93	16.30 ± 114.92	0.75 ± 0.97	ns
SVT	1.33 ± 4.66	1.53 ± 4.77	0.00 ± 0.00	0.036
VPC	85.70 ± 241.74	90.44 ± 251.73	55.25 ± 168.96	0.027
VPC pairs	0.84 ± 4.98	0.97 ± 5.35	0.00 ± 0.00	ns
Bigeminy	0.17 ± 0.98	0.19 ± 1.05	0.00 ± 0.00	ns
Trigeminy	0.91 ± 6.45	0.99 ± 6.91	0.42 ± 1.44	ns
Tachycardia	0.49 ± 2.58	0.48 ± 2.49	0.52 ± 3.18	ns
Bradycardia	0.75 ± 7.10	0.87 ± 7.64	0.00 ± 0.00	ns
Pauses >2.5 s	1.20 ± 7.27	1.39 ± 7.80	0.00 ± 0.00	ns

OSA, obstructive sleep apnea; HR, heart rate; SVPC, single premature supraventricular arrhythmias; VPC, ventricular arrhythmias; SVT, supraventricular tachycardia.

**Table 3:** Qualitative parameters of 24-h ECG Holter monitoring in the studied group of patients.

	Whole Study Group	OSA (Group A)	Without OSA (Group B)	*p* A-B
Sinus rhythm	88/98.9	76/98.7	12/100.0	ns
Escape rhythm	1/1.1	1/1.3	0/0.0	ns
AF	2/2.2	2/2.6	0/0.0	ns
VT	4/4.5	2/2.6	2/16.7	ns
Tachycardia (yes/no)	4/4.5	3/3.9	1/8.3	ns
Bradycardia (yes/no)	1/1.1	1/1.3	0/0.0	ns
Pauses >2.5 s (yes/no)	5/5.6	5/6.5	0/0.0	ns
I-degree atrioventricular block	22/24.7	18/23.4	4/33.3	ns
Wenckebach’s II-degree atrioventricular block	3/3.4	3/3.9	0/0.0	ns
Mobitz’s II-degree atrioventricular block	0/0.0	0/0.0	0/0.0	ns
III-degree atrioventricular block	0/0.0	0/0.0	0/0.0	ns
ST-T changes	11/12.3	8/10.4	3/25.0	ns
∑ quantitative changes	1.58 ± 0.81	1.55 ± 0.79	1.83 ± 0.94	ns

OSA, obstructive sleep apnea; AF, atrial fibrillation; VT, ventricular tachycardia.

**Table 4:** Quantitative parameters of 24-h ECG Holter monitoring in the studied subgroups of patients divided by AHI threshold values.

	AHI ≥ 15 (Subgroup A1)	AHI < 15 (Subgroup A2)	*p*	AHI ≥ 30 (Subgroup A3)	AHI < 30 (Subgroup A4)	*p*
HR mean (bpm)	70.35 ± 7.44	66.59 ± 7.94	0.021	70.97 ± 7.65	67.86 ± 7.75	ns
HR min (bpm)	55.62 ± 6.89	52.41 ± 6.60	0.021	56.13 ± 7.24	53.51 ± 6.65	ns
HR max (bpm)	101.49 ± 13.23	104.53 ± 21.76	ns	102.80 ± 14.30	102.58 ± 18.26	ns
SVPC	225.24 ± 714.84	20.24 ± 32.04	ns	252.57 ± 716.91	93.20 ± 475.25	ns
SVPC pairs	22.49 ± 135.83	0.79 ± 1.09	ns	39.33 ± 183.55	1.42 ± 3.19	ns
SVT	1.93 ± 5.56	0.35 ± 0.98	ns	1.97 ± 4.80	1.00 ± 4.28	ns
VPC	107.18 ± 336.96	72.42 ± 159.30	0.018	101.34 ± 285.43	54.93 ± 113.65	0.035
VPC pairs	0.49 ± 2.33	1.41 ± 7.53	ns	0.17 ± 0.46	1.19 ± 6.10	ns
Bigeminy	0.24 ± 1.23	0.06 ± 0.24	ns	0.00 ± 0.00	0.25 ± 1.20	ns
Trigeminy	0.04 ± 0.19	2.32 ± 10.37	ns	0.03 ± 0.18	1.36 ± 7.90	ns
Tachycardia	0.11 ± 0.81	1.12 ± 4.00	ns	0.20 ± 1.10	0.64 ± 3.07	ns
Bradycardia	1.22 ± 9.03	0.00 ± 0.00	ns	0.00 ± 0.00	1.14 ± 8.72	ns
Pauses >2.5 s	1.58 ± 8.86	0.59 ± 3.43	ns	2.87 ± 11.93	0.36 ± 2.60	ns

AHI, apnea-hypopnea index; HR, heart rate; SVPC, single premature supraventricular arrhythmias; VPC, ventricular arrhythmias; SVT, supraventricular tachycardia.

**Table 5:** Qualitative parameters of 24-h ECG Holter monitoring in the studied subgroups of patients divided by apnea-hypopnea index (AHI) threshold values.

	AHI ≥ 15 (Subgroup A1)	AHI < 15 (Subgroup A2)	*p*	AHI ≥ 30 (Subgroup A3)	AHI < 30 (Subgroup A4)	*p*
Sinus rhythm	54/98.2	34/100.0	ns	29/96.7	59/100.0	ns
Escape rhythm	0/0.0	1/2.9	ns	0/0.0	1/1.7	ns
AF	2/3.6	0/0.0	ns	2/6.7	0/0.0	ns
VT	1/1.8	3/8.8	ns	1/3.3	3/5.1	ns
Tachycardia (yes/no)	1/1.8	3/8.8	ns	1/3.3	3/5.1	ns
Bradycardia (yes/no)	1/1.8	0/0.0	ns	0/0.0	1/1.7	ns
Pauses >2.5 s (yes/no)	4/7.3	1/2.9	ns	3/10.0	2/3.4	0.033
I-degree atrioventricular block	14/25.4	8/23.5	ns	10/33.3	12/20.3	0.039
Wenckebach’s II-degree atrioventricular block	0/0.0	0/0.0	ns	0/0.0	0/0.0	ns
Mobitz’s II-degree atrioventricular block	0/0.0	0/0.0	ns	0/0.0	0/0.0	ns
III-degree atrioventricular block	2/3.6	1/2.9	ns	2/6.7	1/1.7	ns
ST-T changes	5/9.1	6/17.6	ns	3/10.0	8/13.6	ns
∑ quantitative changes	1.53 ± 0.74	1.68 ± 0.91	ns	1.70 ± 0.79	1.52 ± 0.82	ns

AHI, apnea-hypopnea index; AF, atrial fibrillation; VT, ventricular tachycardia.

**Table 6:** Results of the regression analysis in the studied group of patients.

**Model for HR Mean**		
	Intercept	Smoking
Regression coefficient	66.400	4.613
SEM of Rc	7.703	1.737
*p* value	0.001	0.010
**Model for SVPC Pairs**		
	AHI	BMI
Regression coefficient	1.736	3.675
SEM of Rc	0.558	2.110
*p* value	0.003	0.046
**Model for SVT**		
	AHI	male gender
Regression coefficient	0.049	−2.077
SEM of Rc	0.024	0.996
*p* value	0.047	0.040
**Model for Pauses >2.5 s**		
	AHI	glucose
Regression coefficient	0.082	0.061
SEM of Rc	0.040	0.020
*p* value	0.043	0.004

AHI, apnea-hypopnea index; HR, heart rate; SVPC, single premature supraventricular arrhythmias; SVT, supraventricular tachycardia.

## Data Availability

The data presented in this study are available upon request from the corresponding author. The data are not publicly available.
